# The Risk of Obstructive Sleep Apnea in Open-Angle Glaucoma Patients

**DOI:** 10.7759/cureus.18699

**Published:** 2021-10-12

**Authors:** Majed Alotaibi, Mohammed Alsubaie, Abdulrahman Alharthi, Akram Alnabri, Abdullah Bormah, Khalid Alafif, Nizar Alhibshi

**Affiliations:** 1 College of Medicine, King Abdulaziz University Hospital, Jeddah, SAU; 2 Ophthalmology, King Abdulaziz University Hospital, Jeddah, SAU

**Keywords:** middle-eastern populations, osa with high bmi, stop-bang questionnaire, open-angle glaucoma, obstructive sleep apnea

## Abstract

Background

This study aimed to assess the risk of obstructive sleep apnea (OSA) in glaucoma patients using the STOP-BANG questionnaire at the King Abdulaziz University Hospital (KAUH), a tertiary care center in Saudi Arabia.

Methodology

This study used a cross-sectional telephone survey. Patients older than 18 years diagnosed with open-angle glaucoma, without a diagnosis of respiratory disease or steroid use, completed the STOP-BANG questionnaire, a validated tool to determine the risk for developing OSA. Patients with a score of 3 or more were considered at intermediate risk of OSA, and those with a score of 5 or more of the maximum 8 points were considered to have a high risk for moderate/severe OSA. Social demographic information and medical histories were collected from all patients using the medical record system of the KAUH.

Results

A total of 77 patients with glaucoma were included in the study. The mean STOP-BANG score was 3.40 ± 1.5; 27.3% of the patients had low risk of OSA, 36.4% had intermediate risk, and 36.4% had high risk. An evaluation of the OSA symptoms found snoring, tiredness, and observed apnea in 29.9%, 36.4%, and 14.3% of patients, respectively. The association between body mass index and STOP-BANG score was significant.

Conclusions

Our analysis and assessment of the association between glaucoma and OSA found no evidence that glaucoma patients are more likely to have OSA or develop more severe OSA than others. Therefore, we do not recommend systematic screening of glaucoma patients for OSA.

## Introduction

Glaucoma is a widespread and serious, progressive optic nerve disease. It includes congenital glaucoma (CG), normal-tension glaucoma (NTG), angle-closure glaucoma (ACG), and open-angle glaucoma (OAG). OAG is the most common type, representing 50% of all cases [1]. Glaucoma can lead to blindness when left untreated because the changes are irreversible [2]. It is the cause of bilateral blindness in 8.4 million people worldwide (4.47 million with OAG and 3.39 million with ACG) [3]. According to a World Health Organization report, glaucoma is the second leading cause of blindness globally and is responsible for 12.3% of cases [4].

Obstructive sleep apnea (OSA) is the condition of repetitive obstruction of the upper airway during sleep, causing recurrent oxygen desaturation. The prevalence of OSA appears to be higher among men (17-24%) than women (5-9%) [5]. OSA increases the risk of cardiovascular and neurovascular diseases, morbidity, and mortality [6,7]. The STOP-BANG questionnaire is built to screen for OSA in participants by asking eight (yes/no) questions to determine whether the respondents have mild, moderate, or severe OSA based on their total score. The questionnaire can be completed rapidly and efficiently (usually within one to two minutes) and has a relatively high overall response rate (90-100%) [8]. The questionnaires are validated in different populations, with 94% sensitivity and 75% negative predictive value in detecting moderate-to-severe OSA in a sleep clinic setting [9].

Although well reported, the association between glaucoma and OSA as a risk factor is inconclusive. While many studies have associated OSA with glaucoma in general [10-15] and NTG in particular [12,16], with higher sleep disturbance in glaucoma groups compared to the control group, which may be attributed to the association between retinal ganglion cells and the circadian rhythm [16], others have found no such association between glaucoma and OSA [17-20]. Nonetheless, studies on the Middle-Eastern population are limited, and most studies search for glaucoma in patients with pre-existing OSA. Our study aims to assess the risk of OSA in primary OAG patients using the STOP-BANG questionnaire in a tertiary care center in Jeddah, Saudi Arabia, and provide insights into the potential effects of OSA on glaucoma care.

## Materials and methods

Study design and population

We performed a cross-sectional telephone survey using the STOP-BANG questionnaire to assess the risk of OSA in OAG patients. Our target population included patients diagnosed with primary OAG at the King Abdulaziz University Hospital (KAUH) in Jeddah, Saudi Arabia. We included all OAG patients 18 years or older. Patients with respiratory diseases and those using systemic or topical steroids were excluded from the study.

Data collection

Social demographic information and medical history, including the patient’s name, age, sex, weight, height, medical history, admission date, and telephone number, were collected from the medical records system of the KAUH. From January 2015 to August 2020, we called 180 patients using their phone numbers, and 77 were interviewed via telephone using the validated STOP-BANG questionnaire.

STOP-BANG questionnaire

The STOP-BANG questionnaire is used to assess the risk of OSA (Table 1). It has been validated in several populations and has been used frequently in sleep and surgery clinics [21]. It includes eight yes or no questions related to the clinical symptoms and signs of OSA (tiredness, snoring, high blood pressure, observed apnea, age >50 years, body mass index [BMI] <35 kg/m^2^, neck circumference (>41 cm in women, and 43 cm in men), and male gender). The “yes” and “no” responses receive scores of 1 and 0, respectively, and the overall score ranges from 0 to 8. Based on the overall score, patients can be classified as having mild, moderate, or severe OSA. Patients with scores of 0-2 and 5-8 are classified as having a low and high risk, respectively, for moderate-to-severe OSA. However, additional criteria are required to classify patients with mid-range (3 or 4) STOP-BANG scores. For instance, a score of ≥2 and a BMI >35 kg/m^2^ would classify the patient as having a high risk for moderate-to-severe OSA [9].

**Table 1:** STOP-BANG questionnaire and scoring system Proprietary to the University Health Network. Dr. Frances Chung. www.stopbang.ca.

Questions	Yes	No
1. Snoring: Do you snore loudly (loud enough to be heard through closed doors or your bedpartner elbows you for snoring at night)?		
2. Tired: Do you often feel tired, fatigued, or sleepy during the daytime (such as falling asleep during driving or talking to someone)?		
3. Observed: Has anyone observed you stop breathing or choking/gasping during your sleep?		
4. Blood pressure: Do you have or are you being treated for high blood pressure?		
5. BMI: Is your body mass index more than 35 kg/m^2^?		
6. Age: Are you older than 50 years?		
7. Neck collar size of the shirt: S, M, L, XL		
7A. For men, is your shirt collar size (L) 17 inches/43 cm or larger?		
7B. For women, is your shirt collar size (L) 16 inches/41 cm or larger?		
8. Gender: Male?		
Scoring criteria		
Low risk of OSA: Yes to 0–2 questions		
Intermediate risk of OSA: Yes to 3–4 questions		
High risk of OSA: Yes to 5–8 questions		
or Yes to 2 or more of 4 STOP questions + male gender		
or Yes to 2 or more of 4 STOP questions + BMI >35 kg/m^2^		
or Yes to 2 or more of 4 STOP questions + neck circumference (17 inches/43 cm in men, 16 inches/41 cm in women)		

Data analysis

The data were entered using Google Forms and transferred to Microsoft Excel. Data analyses were performed using the Statistical Package for Social Science (SPSS) software, version 23.0 (IBM Corp., Armonk, NY, USA). Categorical variables, including primary variables, were described using frequencies, whereas continuous variables were presented as mean ± standard deviation (SD) or median depending on the distribution. Normal distribution was assessed using histograms and the Shapiro-Wilk test. A Spearman’s correlation coefficient test was used to explore the relationship between BMI and the STOP-BANG scores. The prevalence was expressed as the percentage with a 95% confidence level. Significance was set at p < 0.05.

Ethical approval

Prior to data collection, ethical approval was obtained from the Faculty of Medicine Research Ethics Committee of KAUH, King Abdulaziz University, Jeddah, Saudi Arabia (Reference number 186-20). During data collection, informed oral consent was obtained from each participant. The checklists used in data collection were anonymous, and confidentiality of data was assured.

## Results

A total of 180 phone calls were made to glaucoma patients using their phone numbers obtained from medical records. While 77 (42.8%) patients responded and agreed to participate, 56 did not answer, 34 refused to participate, and 13 were deceased. The demographic characteristics of the patients are shown in Table 2. The mean age of 77 participants was 60 ± 15.3 years, and 67.5% were men. The mean BMI was 27.6 ± 6.4 kg/m^2^, and 7.8% of the patients had a BMI of >35 kg/m^2^. Hypertension was seen in 50.6% of the patients.

**Table 2:** Demographic characteristics of patients. BMI: body mass index

N = 77		%	n
Sex	Male	67.5	52
	Female	32.5	25
Diabetes		7.8	6
Hypertension		50.6	39
BMI >35 kg/m^2^		7.8	6
Age (mean ± SD)		60 ± 15.3	
BMI (mean ± SD)		27.6 ± 6.4	

STOP-BANG questionnaire results

The results of the questionnaire are shown in Table 3. The mean STOP-BANG score was 3.40 ± 1.5. While 27.3% of the patients were at low risk, 36.4% were at intermediate risk, and 36.4% were at high risk for OSA. Regarding OSA symptoms, snoring, tiredness, and observed apnea were reported in 29.9%, 36.4%, and 14.3% of the patients, respectively.

**Table 3:** Results of the STOP-BANG questionnaire. OSA: obstructive sleep apnea

Mean STOP-BANG score	3.40 ± 1.5
Patients with a low risk for OSA (%)	27.3 (n = 21)
Patients with an intermediate risk for OSA (%)	36.4 (n = 28)
Patients with a high risk for OSA (%)	36.4 (n = 28)
OSA symptoms (%)	
Snoring	29.9 (n = 23)
Tiredness	36.4 (n = 28)
Observed apnea	14.3 (n = 11)

## Discussion

The relationship between OSA and glaucoma has been debatable. While some studies have reported a relationship [1-8], others [10-12] have denied the existence of any relationship between OSA and glaucoma. We assessed the risk of OSA in patients with primary OAG using the STOP-BANG questionnaire.

Our results show that the mean STOP-BANG score for glaucoma patients was 3.40 ± 1.5, representing a moderate OSA risk, which is consistent with previous reports [6,13]. The mean STOP-BANG score in our study population (3.40) was comparable to the mean score reported (3.01) for glaucoma patients in an earlier study [14] that assessed the risk of OSA in patients with OAG (versus controls) using the STOP-BANG questionnaire. However, compared to the earlier study, we found a lower proportion of patients with snoring (48.5% vs. 29.9%) and tiredness (48.2% vs. 36.4%), but a higher proportion with observed apnea (8.6% vs. 14.3%). Furthermore, we demonstrated no significant difference in the mean STOP-BANG scores for our study population (3.40) and the control group in the earlier study (3.03), indicating that patients with glaucoma do not show an increased risk for OSA compared to those without glaucoma.

Study limitations

The main limitations of our study were the small sample size, cross-sectional design, and the use of a telephonic questionnaire to assess the potential risk for OSA. Furthermore, because the STOP-BANG questionnaire only estimates the risk of OSA, polysomnography must be performed to assess whether a patient suffers from OSA. Therefore, future longitudinal studies with larger sample sizes are needed to further explore this relationship.

## Conclusions

Our analysis and assessment of the association between glaucoma and OSA found no evidence that glaucoma patients are more likely to have OSA or more severe OSA than others. Therefore, we do not recommend the systematic screening of glaucoma patients for OSA. However, our study was conducted on only a few candidates from a limited population; further studies with larger sample sizes are required to substantiate our findings. Moreover, the STOP-BANG questionnaire could be subjective, only estimating the risk of OSA. Polysomnography would provide more objective and accurate results.
